# Sensitivity analysis to explain the excitability in a pyramidal neuron with application to Alzheimer’s disease

**DOI:** 10.1186/1471-2202-12-S1-P342

**Published:** 2011-07-18

**Authors:** Jakub Nowacki, Hinke M Osinga, Jon T Brown, Andrew D Randall, Krasimira Tsaneva-Atanasova

**Affiliations:** 1 Bristol Centre for Applied Nonlinear Mathematics, Department of Engineering Mathematics, University of Bristol, Queen’s Building, University Walk, Bristol BS8 1TR, UK; 2 Pfizer Applied Neurophysiology Group, MRC Centre for Synaptic Plasticity, School of Physiology and Pharmacology, University of Bristol, University Walk, Bristol BS8 1TD, UK

## 

Intrinsic excitability is one of the pillars of neuronal behaviour . Combined experimental and modelling studies of neuronal excitability often provide an important insight into the brain functions. In this work we analyse a unified model that we derived for CA1/3 pyramidal neurons in Hodgkin-Huxley formalism [1]. We explore the variations of the model behaviour through parameter sensitivity analysis. Model validation against the experimental current clamp data shows that our model reproduces the behaviour of pyramidal cells very well. A characteristic feature of CA1/3 pyramidal cell response is a higher frequency of the first spike pairs. We define an excitability measure that quantifies parameter sensitivity in our model and takes into account this unique feature of the response.

**Figure 1:**
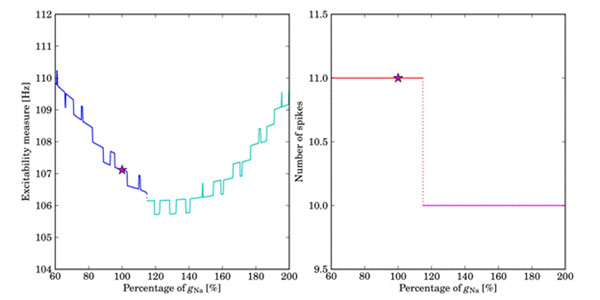
Sensitivity analysis of the maximal conductance of the combined Na+-currents; panel (a) shows the excitability measure ranging over the given percentages of the maximal conductance of Na+-currents; the original value of the maximal conductance is marked by a (magenta) star.

The analysis shows that the outward currents have a considerable influence on both excitability and the number of action potentials. An increase of high-voltage activated inward currents often decreases excitability, whereas an increase of low-voltage activated inward currents results in a large increase of it. Moreover, the outward currents in our model have a profound impact on the number of action potentials. Counter-intuitively, we find that either a decrease or increase of total Na+ current can result in an increase of excitability, as shown in Fig. 1
